# Interaction force modeling and analysis of the human–machine kinematic chain based on the human–machine deviation

**DOI:** 10.1038/s41598-023-43115-9

**Published:** 2023-10-13

**Authors:** Xin Zhou, Zhisheng Duan

**Affiliations:** Department of Mechanics and Engineering Science, Center for Systems and Control, Peking University, Beijing, 100871 China

**Keywords:** Biomedical engineering, Mechanical engineering

## Abstract

A mechanical model for a human–machine interaction force based on the man–machine kinematic chain is established. This is combined with screw theory and a virtual rigid body model for the human–machine interaction force is proposed. This model interprets the basic principle model of the human–machine contact force. The deviation of the human–machine kinematic chain is calculated using the virtual model. In order to carry out the calibration simulation for the model, a 6-sps parallel mechanism is taken as an example to illustrate the construction principle of the human–machine interaction virtual rigid body model. This is calibrated by introducing finite element software. Finally, using the knee exoskeleton as an example, a numerical simulation is introduced. This illustrates the relationship between the driving force of the exoskeleton, the human–machine deviation as well as the virtual stiffness. The modeling method of this paper provides theoretical reference for controller design of human–machine interaction forces in the future.

## Introduction

When controlling a rehabilitation exoskeleton, the object that is directly controlled is the exoskeleton itself, but the ultimate goal is to serve the wearer. Therefore, the interaction between the wearer and the exoskeleton must be taken into account when constructing the control strategy. However, the basic principle of the interaction between the wearer and the exoskeleton is not easy to obtain. The human–machine interaction is mainly concentrated at two levels, physical interaction and cognitive interaction^1^. By identifying the intention of the wearer from different interaction levels, design of a corresponding control strategy is the basis for realizing cooperative control between the wearer and the exoskeleton^2^. Based on the different ways of obtaining the intention of the wearer, the wearer-exoskeleton cooperative control strategy takes human–machine interaction into consideration^3^. It also includes a control strategy based on cognitive interaction^4,5^ and physical interaction^6,7^. In principle, the control strategy based on cognitive interaction can effectively avoid excessive interaction with the exoskeleton. However, the difficulty lies in how to obtain the active intention of the wearer from complex and uncertain EEG or EMG signals. The acquisition of wearer intention based on physical interaction lags behind that of cognitive interaction, but its stability is higher than that of intention recognition based on cognitive interaction. The core of the active intention recognition for the wearer based on physical interaction is the human–machine interaction force between wearers and exoskeletons. However, in view of its complexity during the process of motion, research in this field is still in its infancy^8^. Therefore, it is necessary to establish a model for the human–machine interaction force. Since the existing dynamic model of the human body or exoskeleton cannot fully reflect basic principles of human–machine interactive behavior, the author believes that it is necessary to establish a mechanical model which interprets the interaction principle between the human body and exoskeleton based on the human-machine deviation.

The physical human–machine interaction of the exoskeleton mainly includes the human–machine interface^9^, the human–machine deviation^10^. The human–machine interface is the starting point and foundation of research. Literatures^11–13^ focus on the enabled technology of human–machine interactions based on the human–machine interface. Extensive research including the consistency of human–machine motion as well as the comfort and safety of human–machine connection mechanisms has been carried out. In addition, the human–machine interaction motion and dynamic relationship^12^ need to be further understood. This is so that the external skeleton of human–machine interaction behavior adapts to human motion characteristics and environmental characteristics^13^. The experimental result for interaction energy between human and exoskeleton shows that reducing human energy consumption in a human–machine system does not necessarily increase the exoskeleton mechanical energy output. Overall system performance is greater than the sum of individual partial system performance due to human adaptive ability in a human–machine interaction system^14^. An optimal solution for human–machine interaction control is important for improving the human–machine compatibility of the exoskeleton. Some studies on human–machine interaction force control show that when a wearer is in a systematic interference force field, the original motion mode can be restored^15^. This is done by adaptive torque at the human joint to compensate for external interference^16,17^. When the interference force is suddenly removed, the wearer will make mistakes due to adaptation. Therefore, it is necessary to explore the compensation mechanism and learning mechanism of viscoelasticity for neuromuscular systems^18^. Objects that interact with the wearer produce different spatio-temporal characteristic forces, such as gravity, elasticity and acceleration^17^. These can be compensated for in a dynamic environment. The dynamics of the neuromuscular system can be taken as a feedback mechanism to overcome internal and external interference such as the internal dynamics of human mass or the moment interference between segments. However, since the mathematical complexity of the method, it is difficult to apply it to the actual human–machine interaction system. In order to conform to the characteristics of human–machine interaction, it is necessary to construct an in-depth study of contact force model. During the process of human–machine cooperative movement, the robots confront the contact-impact phenomenon frequently. Therefore, some researchers express the contact forces generated by the contact-impact event as a function of the penetration depth by smoothing the discontinuity of the impact force based on Hertz’s law^19–21^. Literature^22^ introduces a plastic winkle model of plastic conformal contact, and integrate it into a contact solver to simulate the impact dynamics of a single journal-bearing system. In addition, since a collision between two bodies is a usual phenomenon during the human–machine cooperation motion, literatures^23–25^ present the corresponding contact models of the mechanical system. Literature^26^ introduces a model can be used in the hard and soft impact problems, which is completely suitable for the whole range of the coefficient of restitution. However, these mathematical models are too much complex and too many constraints, and the single dimensional contact force cannot reflect the basic principles of human–machine interaction, so the above methods are difficult to meet the real-time requirements of human–machine collaborative motion. Literature^27^ introduces a 6-DOF hand-held force feedback operator with a serial mechanism, by replacing part of the series structure with a parallel mechanism, the overall stiffness performance can be improved. Literature^28^ introduces a combined 6-DOF force feedback device with a sustained force feedback device. However, the existing multi-dimensional force feedback devices are not compact enough and the models are too complex, as a result cannot be applied to the human–machine interaction scenarios. Literature^29^ proposes a capacitive flexible pressure sensor. When the sensor is stressed, the distance between the electrodes is changed through the bending deformation of the inclined micro-column, thus the sensitivity of the sensor can be improved. A flexible piezoresistive pressure sensor is proposed in literature^30^, the sensitivity of the sensor can be improved by designing of gradient nesting structure. Both of the models are relatively simple and easy to implement. However, the existing sensors can only detect a single dimension of the contact force, which is difficult to ensure the multi-dimensional detection requirement of human–machine interaction motion. Compared with the existing human–machine contact force measurement methods, the sensor proposed in this paper is small in size, convenient to carry, and can test multi-dimensional force, and the model establish the mapping relationship between the driving force of the exoskeleton and the interaction force, which is very suitable for the scenario of human–machine collaboration.

In this paper, the knee joint and exoskeleton are integrated into a kinematic chain to form a human–machine closed loop system. The uncontrollable interaction force caused by the human–machine position deviation affects the range of human–machine compatible motion. It also prevents the exoskeleton from providing effective assistance to the human body. The interaction force makes it difficult to control the exoskeleton and hinders the effective completion of human–machine cooperative tasks. In serious cases, it even endangers the wearers^31^. At present, the problem of human–machine deviation is mainly solved by optimizing the size of the exoskeleton structure^32^, introducing flexible connections^33^ or adding passive degrees of freedom^34^. However, the calculation method for the human–machine interaction force has not been described in much detail.

Therefore, it is necessary to establish a mechanical model by considering human–machine coupling behavior. The structure of this paper is as follows: First, a mechanical model for human–machine interaction is established based on the human-machine deviation. Second, by combining with screw theory, a virtual rigid body model of human–machine interaction is proposed. An interaction force model of the human–machine contact force is constructed. In addition, a 6-sps parallel mechanism is introduced into the human–machine contact model, which explains the construction of the virtual rigid body model for human–machine interaction. Moreover, a calibration method is introduced by using finite element software. Finally, the method mentioned above is illustrated using a numerical simulation.

## Establishment of the human–machine interaction model based on the principle of virtual work

**Figure 1:**
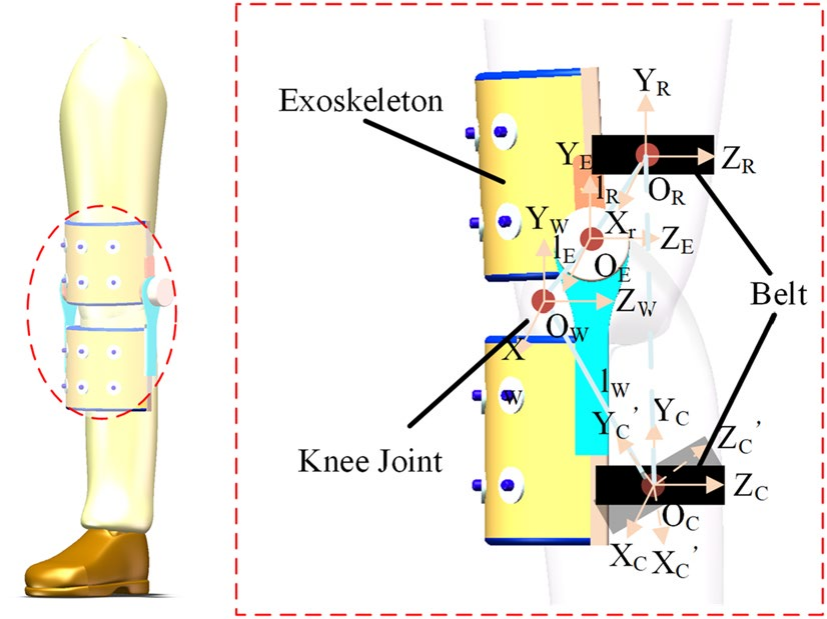
Schematic of coordinate system of the human–machine kinematic chain.

There are many descriptions for human motion in the study of human biomechanics^35^. One view is beneficial for human–machine interaction control. This postulates that the human joint model is equivalent to the rigid body motion model based on the human–machine deviation model. The exoskeleton rigid model is then combined with a human–machine integrated kinematic chain as shown in Fig. 1.

In order to realize the human-machine cooperative motion, the configuration description of the  *e* chain should be equivalent to the *w* chain. Thus, the human-machine closed-chain can be established according to the geometric constraints during the process of human-machine cooperative motion. Figure 1 is a schematic diagram of a human–machine closed-chain for the knee joint and the corresponding human–machine kinematic chain considering interaction deviation. In Fig. 1, R is the reference coordinate system, W is the wearer joint coordinate system, E is the exoskeleton joint coordinate system and C is the human–machine terminal connection point coordinate system. *θ*_*W*_ denotes the set of human motion joint angle variables, *ε* denotes the set of human body deviation variables and *θ*_*E*_ denotes the set of exoskeleton joint angle variables. Since the consistency of geometric constraint relationship in human-machine cooperative motion, the D-H transformation of the wearer joint kinematic chain w is equal to the D-H transformation of the exskeleton chain e, thus the geometric constraint relationship can be obtained:

1 $${}_{C}^{R} T^{E} \left( {\theta_{W} } \right) = {}_{C}^{R} T^{W} \left( {\theta_{E} } \right)$$

In order to accomplish human–machine coupling cooperative motion, the mapping relationship between the human rotation angle of the wearer, the deviation of the human-machine kinematic chain and the rotation angle of the exoskeleton joint can be obtained based on the above equation:

2 $${\mathbf{f}}\left( {\theta_{E} } \right) = \left( {\theta_{W} ,\varepsilon } \right)$$

Moreover, in order to ensure the continuity of the human-machine kinematic chain during the motion process, the following hypothesis needs to be satisfied: Hypothesis 1: The continuity of Eq. (2) should be ensured and the singularity point of the kinematic chain must be avoided. When *f* is an irreversible matrix, the motion singularity will occur. Therefore, the condition of singular configuration is not considered in our equation. For the human–machine closed-loop kinematic chain, the motion of human joints is divided into two parts, namely rigid motion and human–machine deviation motion, which can be expressed as:

3 $${W}\,{\text{chain}}:{{\varvec{\uptheta}}}_{W} = {\mathbf{g}}\left( {\theta_{Er} ,\theta_{Ef} } \right)$$

4 $${E}\,{\text{chain}}:{{\varvec{\upvarepsilon}}} = {\mathbf{h}}\left( {\theta_{Er} ,\theta_{Ef} } \right)$$

where $${{\varvec{\uptheta}}}_{R} = \left[ {\begin{array}{*{20}c} {\theta_{{E{\text{r}}}} } & {\theta_{Ef} } \\ \end{array} } \right]^{T}$$, *θ*_*Er*_ denotes the rigid angle generated by the exoskeleton which mimics the rotation of the human joint. *θ*_*Ef*_ denotes the flexible angle generated by the exoskeleton due to the human–machine deviation. From the above equations, one obtains:

5 $${W}\,{\text{chain}}:{\dot{\mathbf{\theta }}}_{W} = {\mathbf{G}}_{1} \left( {\theta_{E} } \right){\dot{\mathbf{\theta }}}_{Er} + {\mathbf{G}}_{2} \left( {\theta_{E} } \right){\dot{\mathbf{\theta }}}_{Ef}$$

6 $${E}\,{\text{chain}}:{\dot{\mathbf{\varepsilon }}} = {\mathbf{H}}_{1} \left( {\theta_{E} } \right){\dot{\mathbf{\theta }}}_{Er} + {\mathbf{H}}_{2} \left( {\theta_{E} } \right){\dot{\mathbf{\theta }}}_{Ef}$$

This explains the situation during the dynamic interaction of the human–machine kinematic chain. The equations (5) and (6) separate the motion of the human-machine closed-loop chain as a rigid motion and a flexible motion, which is the motion caused by the human-machine deviation. For the closed human–machine kinematic chain, the matrices ***G***_***1***_, ***G***_***2***_, ***H***_***1***_ and ***H***_***2***_ can be regarded as Jacobian matrices of the human–machine kinematic chain. The velocity of the connection point at the end of the  chain and  chain can be expressed as

7 $${\mathbf{J}}_{E} {{\varvec{\uptheta}}}_{E} = {\mathbf{J}}_{W1} {\dot{\mathbf{\theta }}}_{W} + {\mathbf{J}}_{W2} {\dot{\mathbf{\varepsilon }}}$$

Equations (5), (6) is substituted into Eq. (7). The interaction Jacobian matrix of the human–machine kinematic chain can then be obtained as follows:

8 $${\mathbf{J}}_{E} {{\varvec{\uptheta}}}_{E} = \left[ {\begin{array}{*{20}c} {{\mathbf{J}}_{W1} {\mathbf{G}}_{1} \left( {\theta_{E} } \right) + {\mathbf{J}}_{W2} {\mathbf{H}}_{1} \left( {\theta_{E} } \right)} & {{\mathbf{J}}_{W1} {\mathbf{G}}_{2} \left( {\theta_{E} } \right) + {\mathbf{J}}_{W2} {\mathbf{H}}_{2} \left( {\theta_{E} } \right)} \\ \end{array} } \right]$$

Here, the same method is used and it is assumed that the force acting on the terminal of the  chain and the  chain are the same. The mapping relationship between the driving force of the exoskeleton and the human–machine deviation as well as the joint output force of the wearer can be obtained.

9 $${\mathbf{T}}_{Er} = {\mathbf{G}}_{1}^{T} {\mathbf{T}}_{W} + {\mathbf{H}}_{1}^{T} {\mathbf{T}}_{\varepsilon }$$

10 $${\mathbf{T}}_{Ef} = {\mathbf{G}}_{2}^{T} {\mathbf{T}}_{W} + {\mathbf{H}}_{2}^{T} {\mathbf{T}}_{\varepsilon }$$

where ${\mathbf{T}}_{Er}$ and ${\mathbf{T}}_{Ef}$ are the rigid driving force and flexible driving force of the exoskeleton, ${\mathbf{G}}_{1}^{T}$,${\mathbf{G}}_{2}^{T}$, ${\mathbf{H}}_{1}^{T}$, ${\mathbf{H}}_{2}^{T}$ are the Jacobian matrices of the exoskeleton chain and the human body kinematic chain. It is worth noting that Eqs. (9) and (10) contain ***T***_*w*_ and ***T***_*ε*_. ***T***_*w*_ denotes the output force of the wearer joint, which can be measured by the force sensor. ***T***_*ε*_ denotes the human–machine interaction force caused by the human–machine position deviation which is difficult for us to obtain directly. Therefore, a mechanical analysis method of the spatial rigid body is introduced to carry out research on the human–machine interaction force.

## Establishment of the virtual rigid body model of the human–machine interaction

First of all, two basic planes are constructed. One of the planes is fixed to the human skin which is called the skin surface. The other plane is connected to the exoskeleton robot which is called the mechanism surface. The levels between the skin surface and the mechanism surface are composed of several virtual driving branches respectively. Based on this, some assumptions are introduced:

1. Both skin surface and mechanism surface are rigid, so the deformation caused by the human–machine interaction force is transformed into virtual rigid motion driven by each of the driving branches.
2. Since the skin surface, the mechanism surface and the virtual branch have no actual weight, the influence of their inertia forces are ignored.
3. The connection points between the virtual driving branches, the mechanism surface and the points between the skin surface are known.

**Figure 2:**
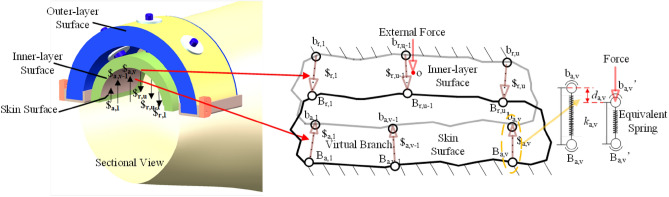
A general human–machine interaction force model.

In this way, the human–machine interaction force is converted into a closed-loop kinematic parallel mechanism. Moreover, the spatial rigid body modeling method is introduced here. A corresponding force analysis method is carried out for this research work. Here, it is assumed that the skin surface, mechanism surface and virtual branches constitute a general parallel mechanism with n DOFs. The forces acting on the mechanism surface are classified into two types. One is the drive force which helps the motion of the mechanism plane, so it is called the driving force. The unit screw of its corresponding driving force is $\user2{\hat{\$ }}_{a,i}$($i = 1, \, 2, \, \ldots , \, m$); the other is the force that restricts the motion of the mechanism plane, which is denoted as restrain force. The unit screw of its corresponding restrain force is $\user2{\hat{\$ }}_{{{\text{r}},j}}$($j = 1, \, 2, \, \ldots , \, 6-n$), without loss of generality (as Fig. 2 shows). Each branch can only apply a single actuated screw or a single constraint screw at the same time.

In order to facilitate the analysis, the reference coordinate system O-xyz is established at the point O of the mechanism plane. The following screws are described in this reference coordinate system as well. If the mechanism surface is affected by the external force *T*_*ε*_ (this is part of the human–machine interaction force and is an external force which is produced by the motion of the exoskeleton and joints of the human body simultaneously) which includes the gravity of the mechanism plane and its inertia force and moment, it can be obtained according to the force balance of the mechanism plane.

11 $$\user2{\$ }_{{\mathbf{F}}} = [\begin{array}{*{20}c} {\user2{\hat{\$ }}_{{{\text{r,}}1}} } & {\user2{\hat{\$ }}_{{{\text{r,}}2}} } & \cdots & {\user2{\hat{\$ }}_{{{\text{r,6}} - n}} } & {\user2{\hat{\$ }}_{{{\text{a}},1}} } & {\user2{\hat{\$ }}_{{{\text{a,}}2}} } & \cdots & {\user2{\hat{\$ }}_{{{\text{a}},m}} } \\ \end{array} ][\begin{array}{*{20}c} {f_{1} } & {f_{2} } & \cdots & {f_{{{6} - n}} } & {w_{1} } & {w_{2} } & \cdots & {w_{m} } \\ \end{array} ]^{{\text{T}}} = {\mathbf{G}}_{{\mathbf{f}}}^{{\mathbf{F}}} {\mathbf{f}}$$

where

$\user2{\hat{\$ }}_{{{\text{r}},j}}$,$\user2{\hat{\$ }}_{{{\text{a}},i}}$: The unit screw of the restrain force $\user2{\$ }_{{{\text{r}},j}}$ and the driving force $\user2{\$ }_{{{\text{a}},i}}$ respectively;

$f_{j}$,$w_{i}$: The magnitude of the restrain force $\user2{\$ }_{{{\text{r}},j}}$ and the driving force $\user2{\$ }_{{{\text{a}},i}}$ respectively;

${\mathbf{G}}_{{\mathbf{f}}}^{{\mathbf{F}}}$: Forward force Jacobian matrix.

${\mathbf{f}}$: A vector composed of the amplitude of the restrain force and the driving force, which can be expressed as:

12 $${\mathbf{f}} = [\begin{array}{*{20}c} {f_{1} } & {f_{2} } & \cdots & {f_{{{6} - n}} } & {w_{1} } & {w_{2} } & \cdots & {w_{m} } \\ \end{array} ]^{{\text{T}}}$$

The external force *T*_*ε*_ can be written as $$T_{\varepsilon } = [\begin{array}{*{20}c} {F_{x} } & {F_{y} } & {F_{z} } & {M_{x} } & {M_{y} } & {M_{z} } \\ \end{array} ]^{{\text{T}}}$$.

In addition, it is assumed that the deformation at the end of each branch is independent, that is, each force screw will not produce deformation in the direction of the other force at the end of the other branch.

Under the action of the driving force ***$***_***a,i***_, the deformation at the end of the branch in the projection direction *d*_*a,i*_ can be obtained:

13 $$w_{i} { = }d_{{{\text{a}},i}} k_{{{\text{a}},i}}$$

where *k*_*a,i*_ is defined as the stiffness of the driving force ***$***_***a,i***_, which is not only related to the stiffness of the branch structure, but also to the axis direction of the driving force.

Similarly, under the action of the restrain force ***$***_***r,j***_, the deformation at the end of the branch in the projection direction *d*_*r,j*_ can be obtained:

14 $$f_{j} { = }d_{{{\text{r}},j}} k_{{{\text{r}},j}}$$

where *k*_*r,j*_ is defined as the stiffness of the restrain force ***$***_***r,j***_, which is also related to the stiffness of the branch structure and the axis direction of the restrain force.

15 $${\mathbf{T}}_{\varepsilon } = {\mathbf{G}}_{{\mathbf{f}}}^{{\mathbf{F}}} {\mathbf{f}} = {\mathbf{G}}_{{\mathbf{f}}}^{{\mathbf{F}}} {\mathbf{Kd}}$$

where $${\mathbf{K}} = diag\left( {\begin{array}{*{20}c} {k_{{{\text{r}},1}} } & {k_{{{\text{r}},2}} } & \cdots & {k_{{{\text{r}},u}} } & {k_{{{\text{a}},1}} } & {k_{{{\text{a}},2}} } & \cdots & {k_{{{\text{a}},v}} } \\ \end{array} } \right)$$.

Previously, it is assumed that both the mechanism surface and the skin surface of the model are rigid bodies. According to the law of energy conservation, the sum of elastic potential energy generated by the driving force and the restrain force is equal to the elastic potential energy generated by the external force. One then obtains:

16 $${\mathbf{T}}_{{{\varvec{\upvarepsilon}}}}^{{\text{T}}} {\mathbf{D}} = w_{1} d_{{{\text{a}},1}} + w_{2} d_{{{\text{a}},2}} + \cdots + w_{v} d_{{{\text{a}},v}} + f_{1} d_{{{\text{r}},1}} + f_{2} d_{{{\text{r}},2}} + \cdots + f_{u} d_{{{\text{r}},u}}$$

where ***D***-Micro-deformation of the mechanism plane generated by the action of external force ***$***_*F*_.

Equation (14) can then be rewritten as:

17 $${\mathbf{T}}_{\varepsilon }^{{\text{T}}} {\mathbf{D}} = {\mathbf{f}}^{{\text{T}}} [\begin{array}{*{20}c} {d_{{{\text{r}},1}} } & {d_{{{\text{r}},2}} } & \cdots & {d_{{{\text{r}},u}} } & {d_{{{\text{a}},1}} } & {d_{{{\text{a}},2}} } & \cdots & {d_{{{\text{a}},v}} } \\ \end{array} ]^{{\text{T}}}$$

By combining Eq. (10) with Eq. (15), one obtains

18 $$[\begin{array}{*{20}c} {d_{{{\text{r}},1}} } & {d_{{{\text{r}},2}} } & \cdots & {d_{{{\text{r}},u}} } & {d_{{{\text{a}},1}} } & {d_{{{\text{a}},2}} } & \cdots & {d_{{{\text{a}},v}} } \\ \end{array} ]^{{\text{T}}} = [{\mathbf{G}}_{{\mathbf{f}}}^{{\mathbf{F}}} ]^{{\text{T}}} {\mathbf{D}}$$

Combining Eqs. (9), (10) and (18) leads to:

19 $${\mathbf{T}}_{Er} = {\mathbf{G}}_{1}^{T} {\mathbf{T}}_{W} + {\mathbf{H}}_{1}^{T} {\mathbf{G}}_{{\mathbf{f}}}^{{\mathbf{F}}} {\mathbf{K}}[{\mathbf{G}}_{{\mathbf{f}}}^{{\mathbf{F}}} ]^{{\text{T}}} {\mathbf{D}}$$

20 $${\mathbf{T}}_{Ef} = {\mathbf{G}}_{2}^{T} {\mathbf{T}}_{W} + {\mathbf{H}}_{2}^{T} {\mathbf{G}}_{{\mathbf{f}}}^{{\mathbf{F}}} {\mathbf{K}}[{\mathbf{G}}_{{\mathbf{f}}}^{{\mathbf{F}}} ]^{{\text{T}}} {\mathbf{D}}$$

**Figure 3:**
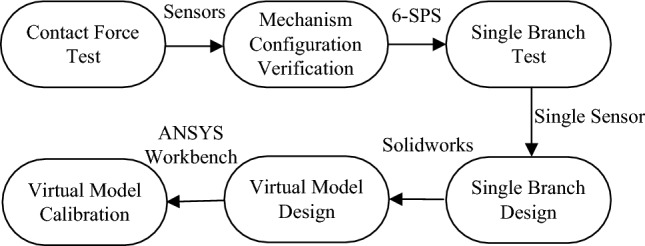
Flowchart of the modeling process.

In this way, a general model of the human–machine interaction force can be established. In the equations above, ***D*** represents the human–machine position and posture deformation under the action of the corresponding human joint driving force and the exoskeleton driving force. Furthermore, the solution for matrix ${\mathbf{G}}_{{\mathbf{f}}}^{{\mathbf{F}}}$ and ***K*** still need to be provided. However, it is noticed that ${\mathbf{G}}_{{\mathbf{f}}}^{{\mathbf{F}}} {\mathbf{K}}[{\mathbf{G}}_{{\mathbf{f}}}^{{\mathbf{F}}} ]^{{\text{T}}}$ in the equation above happens to be the complete virtual stiffness model of the virtual contact force model. Therefore, the contact stiffness model can be calibrated directly. In addition, since the human–machine contact force model is a general model, we can construct different configurations based on what is required to obtain ${\mathbf{G}}_{{\mathbf{f}}}^{{\mathbf{F}}} {\mathbf{K}}[{\mathbf{G}}_{{\mathbf{f}}}^{{\mathbf{F}}} ]^{{\text{T}}}$ directly. This can also be obtained directly from the general calibration method. In this way, the stiffness ***K*** of each branch can be obtained separately. For ease of illustration, a general 6-sps parallel mechanism is introduced. The parallel mechanism has six driving branches and the stiffness of each branch can be considered the same. In addition, the finite element software ANSYS Workbench is introduced to calibrate the actual simulation process as shown in the following figures. For the central point of the mechanism plane, the external force and moment is set as an input. The corresponding displacement of the mechanism plane is set as an output and the whole virtual contact stiffness model is calibrated. The flowchart of the modeling process is shown in Fig. 3:

## Testing of the human–machine contact force

**Figure 4:**
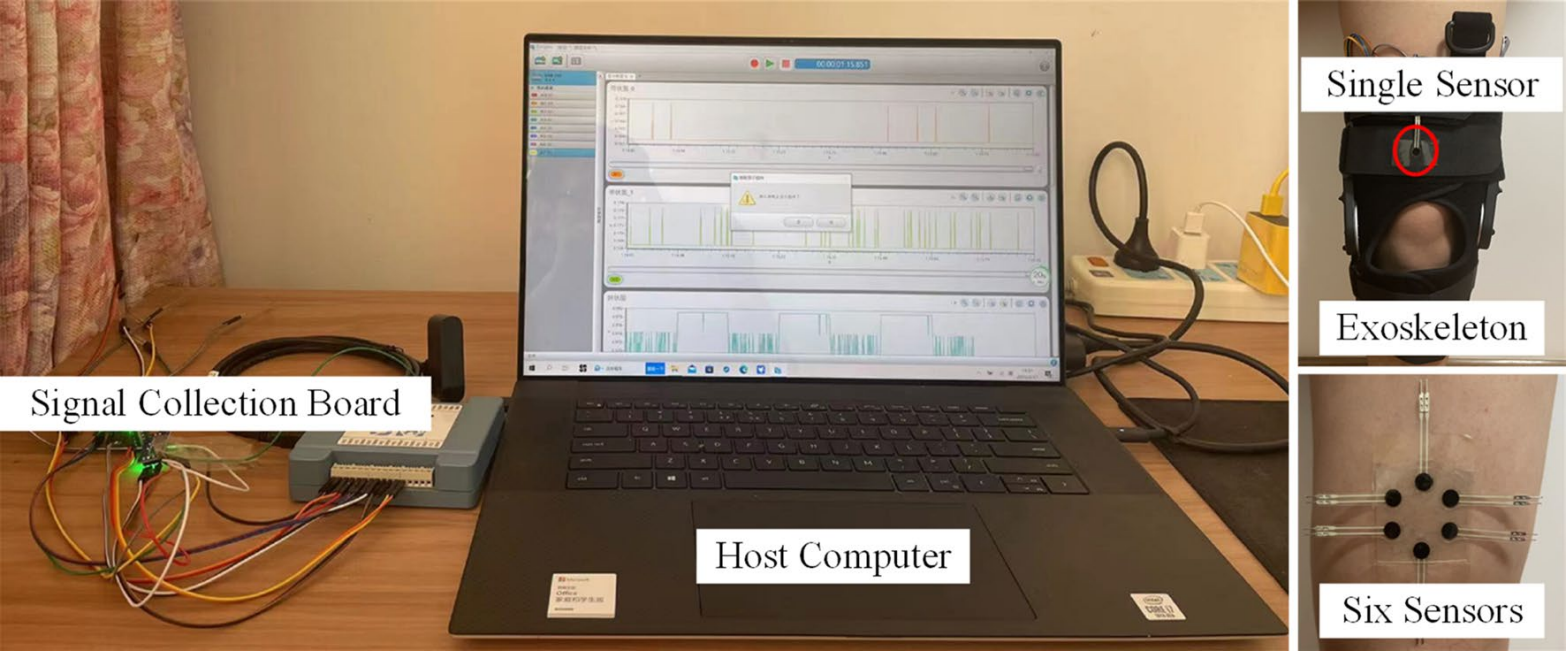
Diagram of the experimental devices.

The experiment devices are shown in Fig. 4. The sensor is amounting on the wearer’s leg, wrapping by the exoskeleton. The signal can be tested by the signal collection board, then transmitted to the host computer. The signal collection board offers eight singled-ended or four differential analog inputs, two analog outputs, 16 digital I/O, and one counter input. The human–machine interface helps users to acquire data and generate signals. The six pressure sensors are arranged uniformly around a circle of radius R_1_, which are underlying the exoskeleton, to make sure that the contact force can be obtained according to the Eq. (15); another pressure sensor is arranged at the center of the circle, which is mounting on the surface of the exoskeleton, to make sure that the external force can be tested.

**Figure 5:**
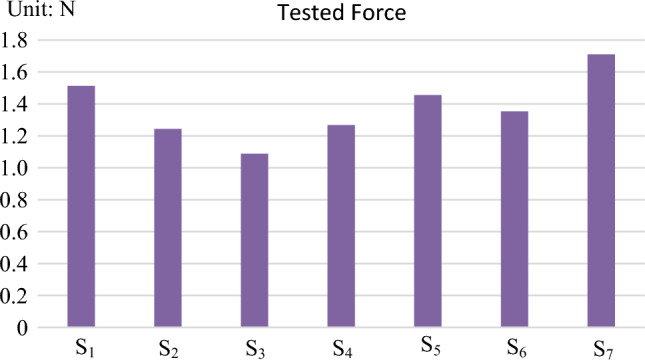
Tested force of each sensor.

The tested force values (average value of 5 times tested) of each sensor are shown in the Fig. 5:

S_1_–S_6_ is the tested force of the sensors mounting on human leg, S_7_ is the tested force of the sensor mounting on the exoskeleton. Thus, the structure parameters of the model can be specified according to the tested force by combing with the virtual contact model.

## Calibration of virtual rigid body model for human–machine interaction

**Figure 6:**
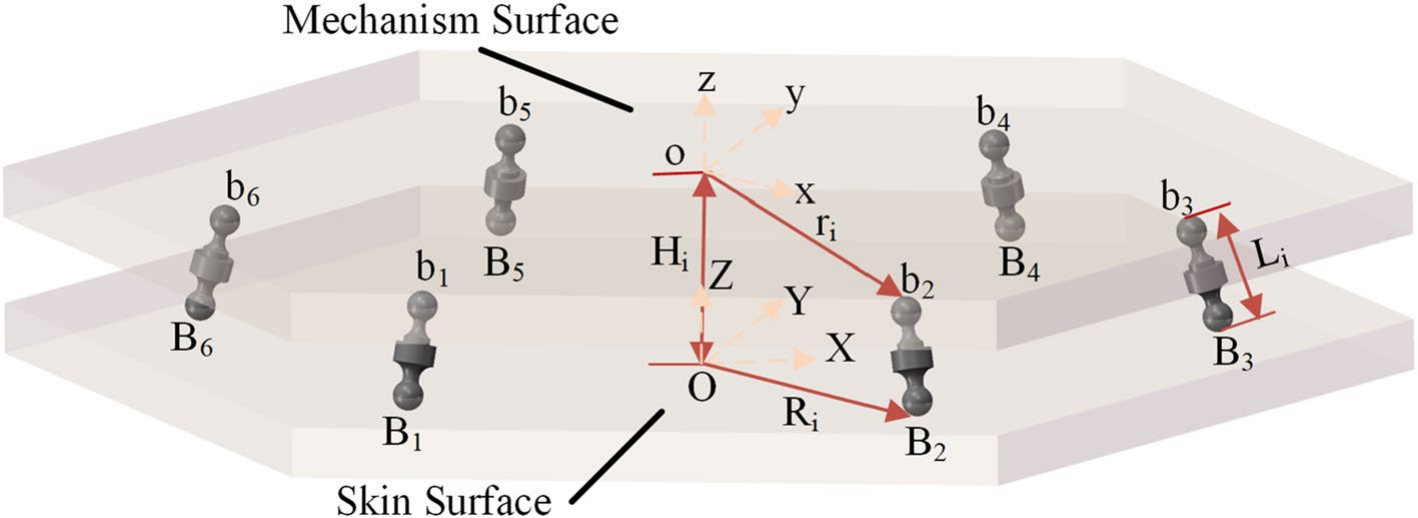
Structural diagram of the 6-sps parallel mechanism.

Next, the 6-sps parallel mechanism is used as an example to illustrate how to realize the virtual rigid body model for human–machine interaction. The structural diagram of the 6-sps parallel mechanism is shown below (see Fig. 6) where the radius of the moving platform (mechanism surface) is r_i_ and the radius of the base (skin surface) is R_i_. The length of the connecting rod is expressed by *L*_i_(i = 1,2…6). By importing the model into the ANSYS Workbench software, the moving platform and base simulate the mechanism surface and skin surface respectively. In the software, the moving platform and base are set to be rigid, and each of the branches is set to be flexible. The specific parameters are shown in the Table 1:

**Table 1:** Mechanical parameters of the virtual model.

Property	Value	Unit	Property	Value	Unit
Density	1020	kg m^−3^	Young’s modulus	2E + 11	Pa
Tensile yield strength	2.5E + 08	Pa	Poisson’s ratio	0.3	None
Compressive yield strength	2.5E + 08	Pa	Bulk modulus	1.6667E + 11	Pa
Tensile ultimate strength	4.6E + 08	Pa	Shear modulus	7.6923E + 10	Pa

After importing the 3D model from Solidworks into ANSYS Workbench, the force and moment are applied in the x, y and z directions respectively for the mechanism face. In order to simulate the contact force between the surface and the mechanism surface, the connections between the joint and two surfaces are set as bonded, the skin surface is set as fixed. Here, the mesh size is decided by combining the mesh quality and experimental comparison, due to limited space, the followings are the briefly introduction of the mesh anaylsis. To improve computing efficiency, the mesh method is set as “Automatic”, the Element Size is set as 2.3 mm, the physics preference is set as “mechanical”. It is worth noting that the accuracy of the model depends on the mesh size, therefore, choosing a small mesh size can reduce the error of the model. However, a smaller mesh size interfere with computational efficiency, thus, we have to choose a reasonable mesh size according to multiple simulations.

In addition, the mechanical parameters of the virtual model are shown in Table 1; the corresponding D–H parameters are shown in Table 2; the constraint condition in the ANSYS Workbench is shown in Table 3. Since the *i*th sps branch consists of six equivalent revolute joints and one prismatic joint, the superscript *j* denotes the number of the corresponding *j*th joint,* a*_*j*−1_,*α*_*j*−1_,* d*_*j*_ and *θ*_*j*_ denote the link length, link twist, link offset and joint angle, respectively. This is to calculate the deformation of the central point of the mechanism surface (linear displacement and angular displacement). Thus, according to the principle of mechanics, the overall stiffness model of the 6-sps mechanism can be obtained.

**Table 2:** D–H parameters of the virtual model.

Number *j*	*a*_*j*−1_ (unit: mm)	*α*_*j*−1_ (unit: degree)	*d*_*j*_ (unit: mm)	*θ*_*j*_ (unit: degree)
1	0	0	0	0
2	0	90	0	90
3	0	90	0	0
4	0	0	2.02	− 90
5	0	− 90	0	90
6	0	− 90	0	0
7	0	− 90	0	0

**Table 3:** Constraint condition in the ANSYS workbench.

Property	Value	Unit	Property	Value	Unit
Analysis system	Static structure	None	Fixed surface	Skin surface	None
Contact	Bonded	None	External force	(1,1,1)	N

**Figure 7:**
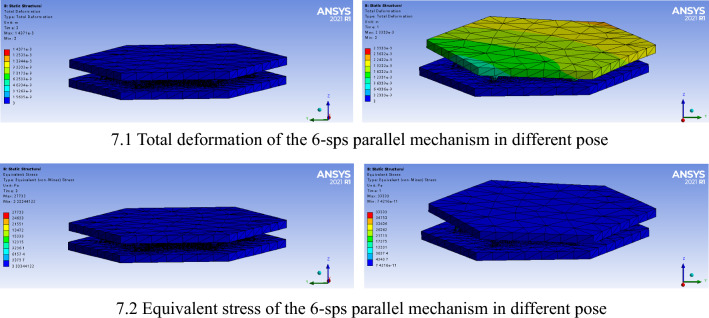
Stiffness performance of the 6-sps parallel mechanism.

**Figure 8:**
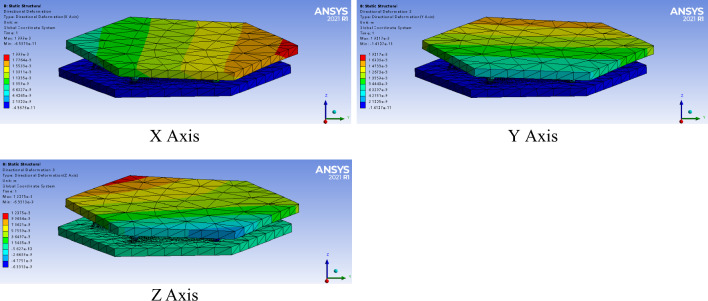
Directional deformation of the 6-sps parallel mechanism in same pose.

Figure 7 shows the stiffness performance of the 6-sps parallel mechanism in different poses. The total deformation denotes the coupling deformation of the mechanism plane under a specific external force. The stress of the 6-sps parallel mechanism is also shown when it is known. For instance, under the initial configuration of the virtual model, the surface deformation is 0.0156–0.1407 mm, and the equivalent stress is 3073.7–27,703 Pa. Under the transformed configuration of the virtual model, the surface deformation is 0.0320–0.2330 mm, and the equivalent stress is 4343.7–39,093 Pa. Obviously, this numerical interval is consistent with the actual situation, which explain the effectiveness of the model, proposed in this paper, to a certain extent. In addition, by observing the cloud map of deformation and stress distribution of the virtual model, it can also provide a reference for investigating the variation and distribution of the interaction force in the process of human–machine interaction. Figure 8 shows the deformation of the mechanism along a specific direction, which can be used to calculate the elements on the primary diagonal of the stiffness matrix.

Through calculating different positions and poses, different groups of solutions can then be obtained. After taking the average value, the overall stiffness of the 6-sps parallel mechanism can be obtained, which can be incorporated into the above equations as a known condition. From Eqs. (19, 20), the transformation relationship between the input force/torque of the exoskeleton and the human–machine position can be established. The knee exoskeleton is then utilized as an example and the change curve of the exoskeleton driving joint is used to solve the relationship between the joint driving force and the deviation.

**Figure 9:**
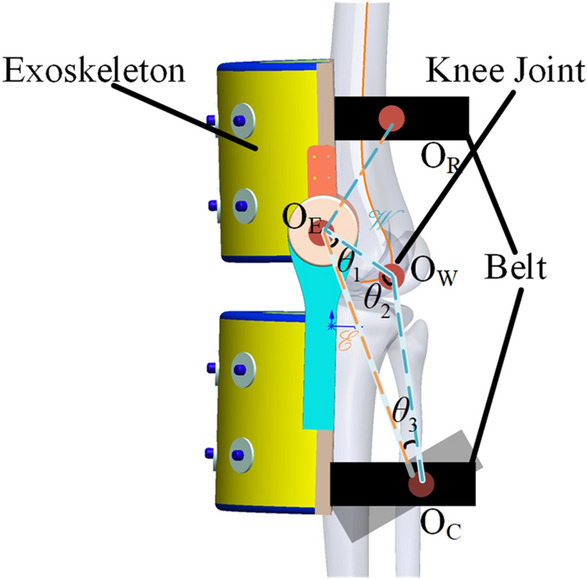
Kinematic constraint model of the human–machine kinematic chain.

If the exoskeleton of the knee joint in Fig. 2 is integrated with the human knee joint to construct the human–machine motion chain (see Fig. 9), the motion principle of the chain can be simplified into the following figure:

where θ_1_ denotes the rotation angle of the exoskeleton, θ_2_ denotes the rotation angle of the wearer knee joint and θ_3_ represents the human–machine rotation deviation. In this way, the motion constraint relationship of the human–machine kinematic chain can easily obtain, that is,

22 $$\theta_{1} + \theta_{2} + \theta_{3} = 180^{ \circ }$$

In addition, since the rotation of the knee and ankle joints of the human body need to be kept synchronized, the following expression can be obtained:

23 $$\theta_{1} = \theta_{2}$$

According to the above calibration method, the virtual rigid body for human–machine interaction can be obtained. Since the equivalent human–machine kinematic chain here is a simplified model, and only one rotational degree of freedom has to be considered, stiffness along other directions in the principal diagonal of the virtual rigid body model is ignored. The corresponding angular stiffness *k* is calculated as 5 Nm/deg through finite element simulation. It is assumed that the human–machine deviation vary in the form of a cosine curve. The variation curve of the joint output torque *T*_*Er*_ and *T*_*Ef*_ of the required exoskeleton is shown in Fig. 9.

**Figure 10:**
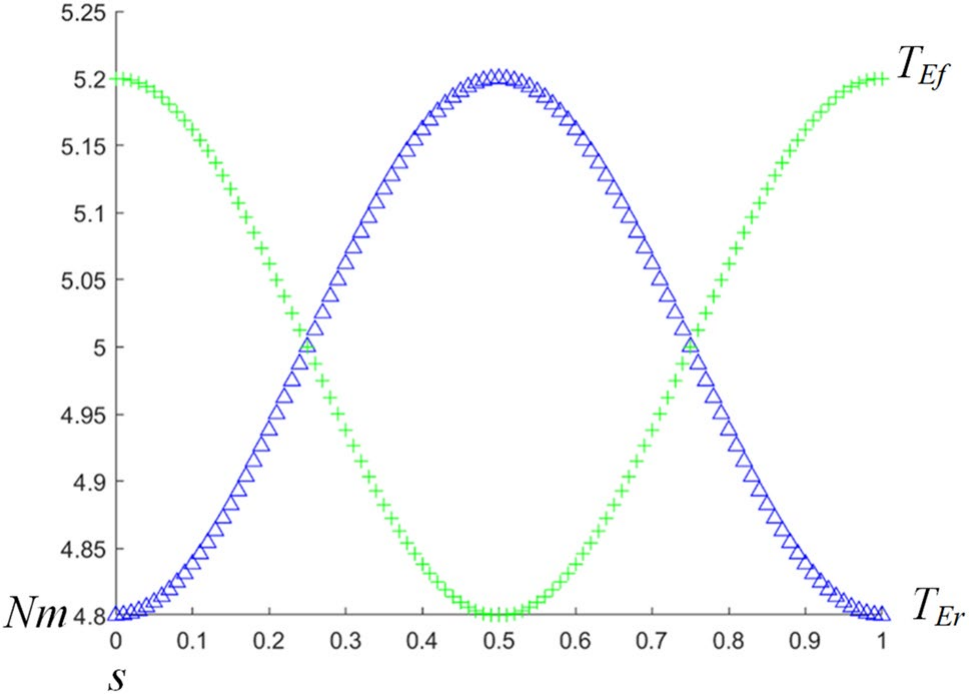
The variation curve of the wearer joint and the exoskeleton output torque.

**Figure 11:**
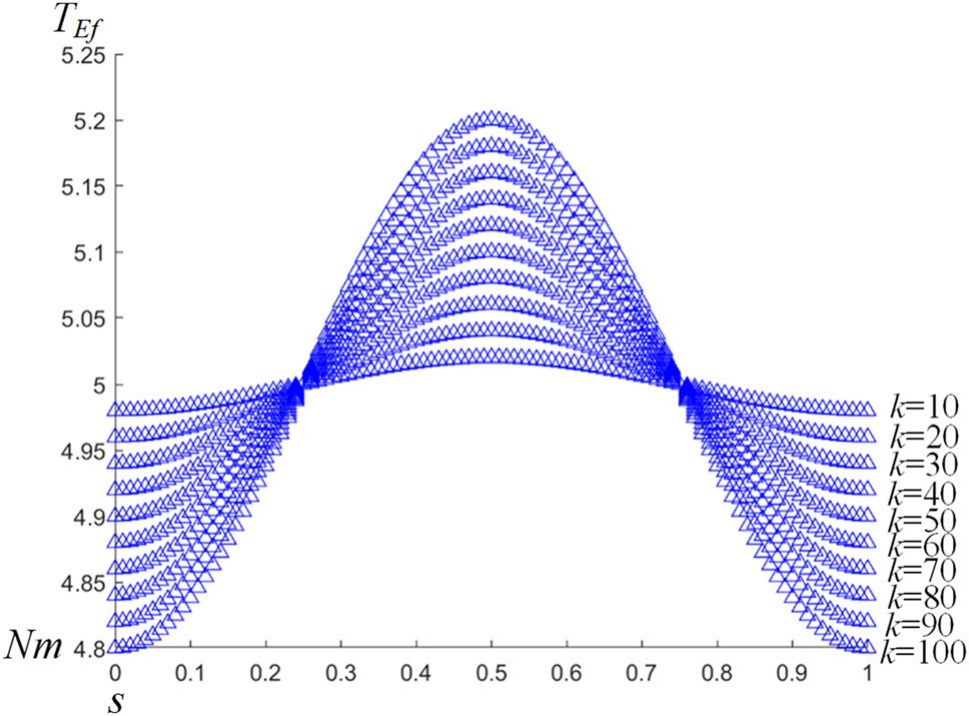
The variation curve of the exoskeleton output torque correspond different stiffness *k.*

As shown in Fig. 10, the joint output torque *T*_*Er*_ and *T*_*Ef*_ of the exoskeleton show a trend of growth and decline respectively. The maximum value (5.2 Nm) of *T*_*Ef*_ occurs twice in one period, when *T*_*Er*_ is the minimum value(4.8 Nm).Therefore, in order to ensure the input torque of the exoskeleton, the torque caused by human–machine deviation should be reduced as far as possible. This will improve human–machine cooperative motion. For *T*_*Ef*_, it can be seen that (Fig. 11) the corresponding stiffness has different effects on it. It is assumed that when the value of D is constant, the change of virtual stiffness has different effects on the amplitude of *T*_*Ef*_: The larger the virtual stiffness *k*, the greater the amplitude change of *T*_*Ef*_. On the contrary, the smaller the virtual stiffness *k*, the smaller the amplitude of *T*_*Ef*_. Therefore, in human–machine interaction design, the material of the human–machine interaction interface should be reasonably selected. This is to reduce the value of the virtual stiffness *k* as much as possible so as to reduce the exoskeleton joint torque output.

## Conclusion

This paper mainly introduces human–machine position and posture deviation. The wearer driving force is separated from the exoskeleton driving force and a mechanical model of the human–machine interaction is constructed. By combining this model with screw theory, this paper proposes a virtual rigid body model for human–machine interaction force. It expounds a method for constructing the human–machine contact force model. Compared with the traditional human–machine interaction force model, the proposed model can well quantify the contact force in six-dimensional space. By introducing the human-machine deviation and the mechanical model of a 6-sps parallel mechanism, the mapping relationship between external force and contact force is effectively established, which provides an effective theoretical basis for improving human–machine interaction force. In addition, A finite element simulation of the 6-sps parallel mechanism is introduced to illustrate the calibration method for the human–machine interaction virtual rigid body model. Finally, a numerical simulation of the knee exoskeleton model is carried out to show the relationship between the virtual stiffness, the driving force of the exoskeleton and the deformation of the human–machine interaction.

However, although the model of human–machine interaction force has been established, some nonlinear factors (such as friction) have not been taken into account, so there are still some errors between the theoretical model and the actual measured values. In the future, we not only need to optimize the mathematical model, but also need to carry out more calibration and experimental work of the contact force model. In addition, the sensor in the paper is more susceptible to disturbance caused by changes in the external environment during the human–machine coordination process. Therefore, we will conduct in-depth research on the stability and robustness of the sensor in the future.

## Data Availability

The data that support the findings of this study are available from the corresponding authors upon reasonable request.
